# Cold agglutinins revealed by abnormalities to the cell blood count: a case report

**DOI:** 10.11604/pamj.2021.38.328.9100

**Published:** 2021-04-06

**Authors:** Hafid Zahid, Rachid Hadef, Faycal Labrini, Anass Yahyaoui, Nezha Messaoudi

**Affiliations:** 1 Laboratory of Hematology and Immuno-haematology Military Instruction Hospital Mohammed V, Rabat, Morocco

**Keywords:** Cold hemagglutinin, haematological abnormalities, temperature range, case report

## Abstract

Cold agglutinin are erythrocyte antibodies which possess the property of agglutinating red blood cells at temperatures of below 37°C, this phenomenon is reversible after heating. This is usually immunoglobulin M (IgM) class. Their pathogenicity is much more related to their temperature range of activity than their title. As we report in this observation, cold hemagglutination makes it difficult to interpret certain immunological tests such as ABO Rh blood grouping or searching for irregular antibodies (SAI). The discovery of cold agglutinins can be fortuitous revealing itself by disturbances and aberrations in the results of blood count or as part of a suggestive clinical or laboratory table cold hemagglutinin disease. The search for a lymphoid hematological at their diagnosis should be systematic.

## Introduction

Cold agglutinin are erythrocyte antibodies which possess the property of agglutinating red blood cells at temperatures of below 37°C but agglutination is best between 0 and 4°C but can be observed up to 20-25°C. This phenomenon is reversible after warming [1]. Most are class immunoglobulin M (IgM), although the IgG or IgA are sometimes found [2]. Cold agglutinins may, in case of significant activation, incidentally interfere with certain laboratory tests. The hemagglutination makes interpretation of ABO Rh blood typing difficult and may mask the presence of alloantibodies in the search for irregular agglutinins (SIA) [3]. They can also affect the results of the blood count. These agglutinins are specifically sought in the context of a suggestive clinical or laboratory table cold agglutinin disease (dermatological assessment, anemia and / or autoimmune haemolysis). Apart from idiopathic chronic cold agglutinin disease, cold agglutinin (CA) can be observed in various circumstances [4]: mainly lymphoid hematological malignancies and infections. Moreover, the serum of normal subjects may contain so-called natural CA, as low (< 1/16) and active only cold, which must be distinguished from CA with real pathological significance [5].

## Patient and observation

It's about a man of 55 years, without significant medical or surgical history, hospitalized for exploration of acute anemic syndrome. The blood count shows anemia to 4.4g/dl of hemoglobin, macrocytic (MCV to 135.1 fL) with an MCH of 87,3g/dl, MCHC a 66.4% and a decrease in the number of red blood cells (500000 /μl). Leukocytes rate (8900 /μl) and platelets (202000 /μl) are normal. The blood smear shows erythrocytes rolls. These hematological constants reveal a false macrocytosis, a false drop in the number of red blood cells, an absurd increase in MCH and MCHC and moving towards the presence of cold agglutinins. Macroscopically, lumps have been observed on the wall of the tube of anticoagulated whole blood (EDTA). The quick recovery of blood count on the same tube after incubation at 37°C for 1 to 2 hours note the correction of anomalies. A biochemical review of hemolysis notes an increase in LDH (1172U / L) and a drop in haptoglobin (0,010g/L) in plasma.

**Figure 1:**
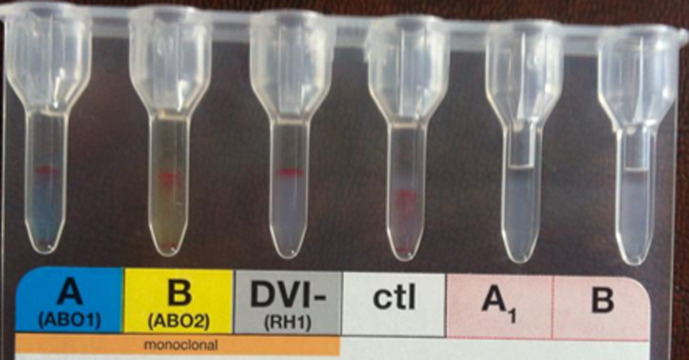
ABORH1 were in-interpretable

**Figure 2:**
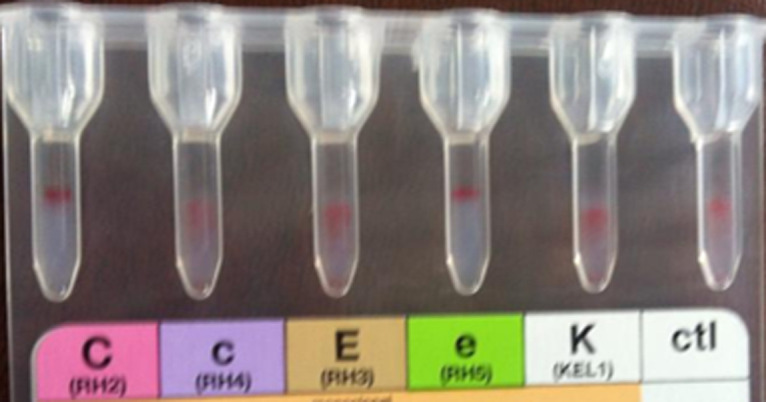
RHKEL1 were in-interpretable

**Figure 3:**
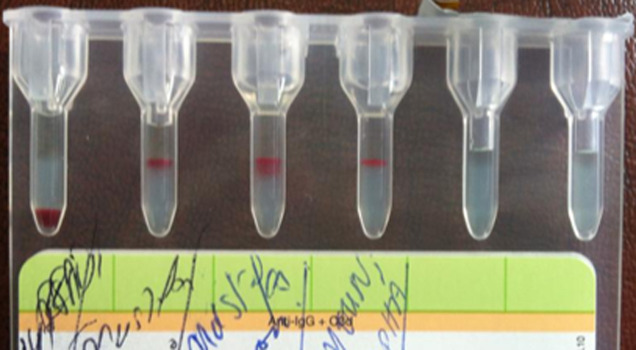
direct antiglobulin test (DAT)

Some immuno-hematological examinations requested in pre-transfusion assessment made on gel card holder such as ABORH1 grouping, RH.Kell1 were in-interpretable due to agglutination in all reactions including those of the controls (Figure 1 and Figure 2). The examinations referred to in etiologic diagnosis of haemolytic anemia such as direct antiglobulin test (DAT) performed at 4°C and 37°C reveals the presence of anti-IgG and anti-C3d (Figure 3) while the indirect antiglobulin test (IAT) is an anti-public suspect whose identification is not required.

## Discussion

Cold agglutinins are rare. Their incidence and prevalence are probably underestimated given the wide variability of clinical manifestations. It is also noted a steady increase in incidence with age mainly after 50 years. Biological investigations have shown that the haptoglobin was collapsed, in the majority of cases of hemolysis, signing intravascular hemolysis.

**Research of cold agglutinin in the laboratory:** the levy is collected in EDTA tube (whole blood) or dry tube (serum). Must be properly made and kept at + 37°C until serum decantation to avoid binding of the antibodies to erythrocytes. The direct antiglobulin test (DAT): detects the presence of cold antibodies bound to red blood cells. The passage of a complement fixing antibodies, only persists fraction C3d on erythrocytes remained intact. The test is positive in 90% of cases of cold agglutinin disease, usually positive type C3d and IgG negative. These antibodies bind to cold but elute at 37°C and therefore remain free in the serum.

Indirect antiglobulin test (IAT) at 4°C: detected negative on serum maintained at + 37°C positive at + 4°C. Titles from 64 to + 4°C are significant and the threshold to 256 is proposed to really assert the presence of pathogenic cold agglutinins. The temperature range is the most important, however. Even in case of low titer at 4°C if cold agglutinin is still active at 30 -37°C, there may be a hemolysis. Cold agglutinins are often directed against public antigens I and sometimes i, precursors of blood group ABH and Lewis. There are also anti-HI, sometimes anti-MNS1 (M) or anti-P1 and rarely anti-A1 or anti-B. This identification is not useful because there are no blood donors negative for these antigens.

### Types of cold agglutinins

**The cold alloantibodies**, the cold antibodies, the IgM type, such as anti-MNS1 (M), anti -P1 or anti-Lea (LE1) are irregular natural antibodies, often less active at 37°C. They are present in very low serum titers in many individuals. They are not necessarily pathogens and rarely responsible for transfusion accidents.

**The cold autoantibodies**, in general, are IgM type, but exceptionally IgG or IgA type are most frequent. The spontaneous agglutination of the sensitized red cells by these cold antibodies is visible on blood smears rollers appearance, sometimes also macroscopically on the anti-coagulated blood tube. It occurs in the superficial vessels of the extremities where the temperature can drop to 28 - 30°C. It causes signs of acrocyanosis and can lead to necrosis of the extremities if ischemia is prolonged. IgM or IgG type of cold antibodies bind to red blood cells, activate complement and generate intra and extravascular hemolysis. Therefore, they are responsible for autoimmune hemolytic anemia (AIHA) known as auto-antibodies cold and represent 16-32% of AIHA. Cold autoantibodies of IgA Nature, incapable of fixing complement, are responsible only of vascular signs. The pathogenicity of these antibodies is more related to their thermal amplitude than their concentration. When the temperature range is low, hemolysis only occurs accordingly in case of cooling.

**Clinical manifestation:** clinical and biological manifestations of cold agglutinins are highly variable and are not always correlated to their biological characteristics (capacity, temperature range, specificity) justifying a biologist clinician consultation in difficult cases [5].

They may be asymptomatic and discovered incidentally. In case of a clinical manifestation, they are responsible for:

**Chronic cold agglutinin disease:** usually occurring in a context of autoimmunity or lymphoid hematological disease such as Waldenstrom or lymphoma in patients over 50 years. It is called pre-neoplastic. The cold agglutinins are IgM monoclonal. Symptoms are often only those with chronic anemia which progresses gradually [6].

**Acute forms:** occurring usually in an infectious context in young adults, adolescents or children (EBC infection, CMV, *Mycoplasma pneumonia*, flu, mumps...). The cold agglutinins are polyclonal IgM. Particular acute form: Paroxysmal hemoglobinuria a frigore, exceptional, observed in children, 8 to 10 days after viral infection (measles, varicella, mumps...). It is related to the transient presence of polyclonal IgG. It can cause severe intravascular hemolytic crises with sudden death.

## Conclusion

The clinical presentation of cold agglutinins is highly variable between life-threatening intra-vascular hemolysis and the absence of any symptoms. Their presence can be suspected by chance due to possible interference on the blood count. Their research is not always obvious. More than just their headline, it is their thermal amplitude activity that is the most important to their pathogenicity. Cold agglutinins are called preneoplastic and the search for a lymphoid hematological is systematic in these patients.
